# Theta-Burst Stimulation Combined With Virtual-Reality Reconsolidation Intervention for Methamphetamine Use Disorder: Study Protocol for a Randomized-Controlled Trial

**DOI:** 10.3389/fpsyt.2022.903242

**Published:** 2022-07-05

**Authors:** Yatong Wen, Xuemin Hao, Xijing Chen, Siyue Qiao, Qianling Li, Markus H. Winkler, Fenglan Wang, Xiaoli Yan, Fang Wang, Liang Wang, Feng Jiang, Paul Pauli, Xinwen Dong, Yonghui Li

**Affiliations:** ^1^Key Laboratory of Mental Health, Institute of Psychology, Chinese Academy of Sciences, Beijing, China; ^2^Department of Psychology, University of Chinese Academy of Sciences, Beijing, China; ^3^School of Education, Shaanxi Normal University, Xi'an, China; ^4^MOE Key Laboratory of Modern Teaching Technology, Shaanxi Normal University, Xi'an, China; ^5^Sino-Danish College, University of Chinese Academy of Sciences, Beijing, China; ^6^Department of Psychology I, Biological Psychology, Clinical Psychology, and Psychotherapy, University of Wurzburg, Wurzburg, Germany; ^7^Shanxi Women's Drug Rehabilitation Center, Taiyuan, China; ^8^Library, Shanxi Medical University, Taiyuan, China

**Keywords:** cue reactivity, drug craving, methamphetamine use disorder (MUD), reconsolidation-based intervention, theta-burst stimulation (TBS), transcranial magnetic stimulation (TMS), virtual reality (VR)

## Abstract

**Background:**

Craving associated with drug-related memory is one of the key factors that induce the relapse of methamphetamine (MA). Disruption or modulation of the reconsolidation of drug-related memory may serve as an option for clinical treatment of MA addiction. This protocol proposes to use virtual reality (VR) to retrieve drug-associated memory and then use transcranial magnetic stimulation (TMS) at the neural circuit that encodes the reward value of drug cues to provide a non-invasive intervention during reconsolidation. We aim to evaluate the effectiveness of TMS treatment after VR retrieval on the reduction of cue reactivity and craving of MA.

**Methods:**

This is a randomized, double-blind, sham-controlled, parallel group trial, targeting participants with MA use disorder aged from 18 to 45 years old. Forty-five eligible volunteers in Shanxi Drug Rehabilitation Center will be recruited and be randomly allocated into three parallel groups, receiving either 1) MA-related cues retrieval in VR combined with active TMS (MA VR scene + TBS) or 2) sham TMS (MA VR scene + sham TBS), or 3) neutral cues retrieval in VR combined with active TMS (neutral VR scene + TBS). Two sessions of post-VR-retrieval TBS will be scheduled on two separate days within 1 week. The primary outcome will detect the memory-related activity by the electroencephalography (EEG) reactivity to drug cues in VR scenes. Secondary outcomes are the self-reported MA craving in VR scene, the physiological parameter (cue-induced heart rate) and the scores of psychological questionnaires including anxiety, depression, and mood. All primary and secondary outcomes will be assessed at baseline, 1-week, and 1-month post-intervention. Assessments will be compared between the groups of 1) MA VR scene + TBS, 2) MA VR scene + sham TBS and 3) neutral VR scene + TBS.

**Discussion:**

This will be the first study to examine whether the TMS modulation after VR retrieval can reduce self-reported craving and drug-related cue reactivity. It will promote the understanding of the neural circuit mechanism of the reconsolidation-based intervention and provide an effective treatment for MA use disorder patients.

**Clinical Trial Registration:**

[Chinese Clinical Trial Registry], identifier [ChiCTR1900026902]. Registered on 26 October 2019.

## Introduction

Methamphetamine (MA) is a potent psychostimulant highly addictive for its euphoric and stimulant effects. It is the most prevalent drug of abuse in China accounting for more than 50% substance users. The number of MA users exceeds one million according to an official survey conducted in 2020 (1) while there is no established therapy for MA dependence (2). A core symptom of MA use disorder (MUD) is craving that lingers even after a long-term abstinence. Craving for MA positively correlates with the severity of MUD (3) and predicts relapse (4). Craving can be induced by cues and contexts associated with drug-related episodic memory (5). The vivid and multi-sensory episodic memory formed through repeated MA use persists after abstinence and drives craving (6), suggesting that intervention on drug-related episodic memory may reduce craving and contribute to relapse-prevention of MUD.

It has been postulated that long-term episodic memories could be modified during a time window called “reconsolidation” after memory retrieval (7, 8). Within the reconsolidation window, memories could be updated, strengthened, or weakened by manipulation of the electrophysiological or neurochemical activity within the neural network encoding the retrieved memory (7, 9). A feature of reconsolidation interventions is that a single or limited numbers of interventions can generate long-term effect for weeks or even months (10–14). Particularly, post-retrieval intervention during reconsolidation reduces the emotional or motivational value of reward cues embedded in episodic memories without disrupting the cognitive component of the cue-reward associative memory (15). Compared to a general reward cue, the motivational value, or incentive salience, of MA cues is stronger and more enduring. Thus, a major difficulty is how to reactivate and destabilize drug-related memory to make it more labile and susceptible to updating. We believe that virtual reality (VR) can reactivate MA-using memory because a vivid, immersive, and interactive virtual scenario carries more contextual information than pictures or videos, which can trigger re-experiencing reward-related memory and potent cravings (16–19). Therefore, we propose that the use of VR to retrieve memory and an MA-craving state provides the possibility for the subsequent intervention.

The reconsolidation of drug-related episodic memory recruits the medial prefrontal cortex and basolateral amygdala (BLA) circuit (20–22) where local pharmacological intervention during reconsolidation can degrade the emotional/motivational impact of the drug-related cues (15). The medial prefrontal cortex is a major cortical center involved in value computation and representation with its multiple sub-regions conducting varied functions (23, 24). The orbitofrontal cortex (OFC) is critical for encoding and updating the incentive value of reward cues in a state-based manner by a reciprocal connection with the BLA (25–27). Clinical studies in substance users found that the OFC dysfunction leads to impaired motives for general rewards but an increased motivation for addictive drugs which gradually develops into a pathological substance craving (28). In addition, inhibition of the OFC or disruption of the OFC-BLA connectivity reduces context- or cue-induced drug seeking (28–31). Although the current evidence supports an idea that the OFC intervention has an immediate effect on cue-induced craving, there is no report on whether inhibition of the OFC can produce a sustained effect to reduce drug-seeking.

Transcranial magnetic stimulation (TMS) is a non-invasive method to modulate neural activity on cerebral cortex by generating a magnetic field close to the scalp over the target brain region (32). TMS with different parameters and stimulation sites can induce long-term changes that facilitate or reduce the activity of neurons or circuits (33). It has been applied as a physical treatment to many neurological and mental disorders such as major depressive disorder (34), obsessive-compulsive disorder (35), and post-traumatic stress disorder (36). Studies with TMS intervention targeting the OFC are rare but their encouraging results show that self-reported cravings for alcohol (28), cocaine (28), or smoking (37) were reduced immediately after OFC or related prefrontal cortex stimulation. Notably, a study on TMS interventions for MA patients compared the effects of stimulation targeting the dorsolateral prefrontal cortex (DLPFC) or ventromedial prefrontal cortex (vmPFC) or both regions for 2 weeks and found that all three treatments reduced MA craving, but TMS over the vmPFC or both yielded a shorter respondence time and a more pronounced effect on sleep quality (38). Another study for alcohol users showed that 10 days of medial prefrontal cortex TBS reduced the connectivity between multiple brain regions in reaction to alcohol cues as well as alcohol drinking up to 3 months after TBS treatment (39). This encouraging report suggests that neuromodulation like TBS on the neural circuit mediating reactivity to addictive drug-cue may achieve a sustained inhibitory effect on drug using.

The purpose of this protocol is to design a set of clinical treatments to obtain a sustainable inhibitory effect on the craving for MA after a limited number of interventions. We propose that TMS stimulation of the OFC within the time window of MA-related episodic memory reconsolidation will produce a continuous reduction in craving elicited by MA-related cues or context. In specific, we will use theta-burst stimulation (TBS), a new pattern of TMS, to modulate the activity in the OFC because of its advantage for stronger effects with shorter duration compared to conventional repetitive TMS (40). We will choose continuous TBS (cTBS) due to its promised effect on depression of cortical excitability (22, 31, 33). It has been delivered to the medial prefrontal cortex to decrease reactivity to alcohol-related cues and reduce drinking behavior in a recent studies (39). And cTBS has been used to disrupt OFC network activities in previous studies (41). Since an effective activation of episodic memory is important in reconsolidation-based treatment (42, 43), a series of vivid and immersive VR scenes related to MA using will be present for memory retrieval and craving self-rating (43–45). In addition to self-report craving, the cue-related electroencephalogram (EEG) activity in the VR scene will be recorded as an indirect measure of the degree of memory retrieval and craving (43, 46, 47). Compared to neutral scenarios, the incentive salience of drug-related cues is stronger to draw drug user's attention (48, 49). Attention bias for drug-related cues is associated with subjective craving and tendency to approach drug-related cues (50, 51), indicated by abnormal EEG activities of prefrontal cortex. For example, two studies reported that MA users showed specific EEG features induced by MA-related scenes presented by VR, such as decreased gamma activity in OFC and right DLPFC (52) and reduced EEG power in delta, theta, and alpha bands (53) comparing to neutral scenario. In addition, processing alcohol- or smoking-related cues coincided with surged frontal beta-band activity in binge drinkers (54) or smoker (55); the increased beta-band activity in response to the drug-related cues is associated with enhanced attention and alertness. A recent study further showed that a higher level of synchronization of medial prefrontal cortex for the beta-band activity in 1–3 months abstinent MUD group, compared to groups with longer or shorter abstinence. Meanwhile, MA craving was higher in the 1–3 months abstinence group than other groups (56). This finding suggests that increased prefrontal beta activity may serve as a robust indicator of MA craving.

In summary, we design a randomized-controlled study of memory reconsolidation-based TBS intervention for people with MUD. It aims to investigate the short-term and long-term effects of TBS treatment on reducing cue-induced craving and updating memory-related EEG reactivity for MUD patients. Meanwhile, the physiological parameter such as cue-induced heart rate and possible co-variables such as emotional and mental state will be considered.

## Methods/Designs

### Study Design

**Figure 1:**
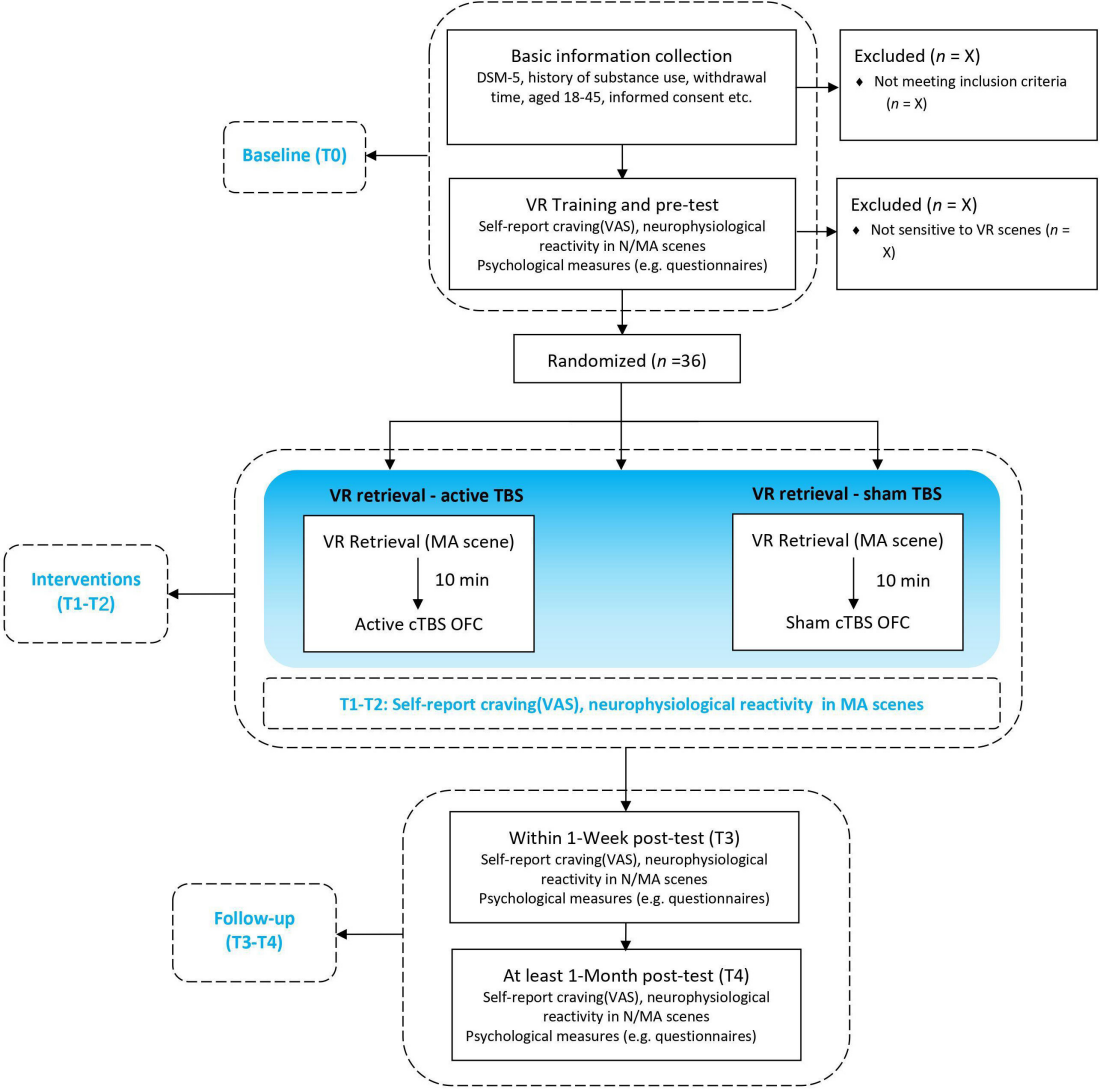
Flow chart of the study design. VR, Virtual Reality; N/MA scenes, neutral/methamphetamine scenes (displayed by VR); cTBS, continuous theta-burst stimulation; OFC, orbitofrontal cortex; neurophysiological reactivity including electroencephalogram (EEG) activities and electrocardiography (ECG) activities in outcome measures.

**Figure 2:**
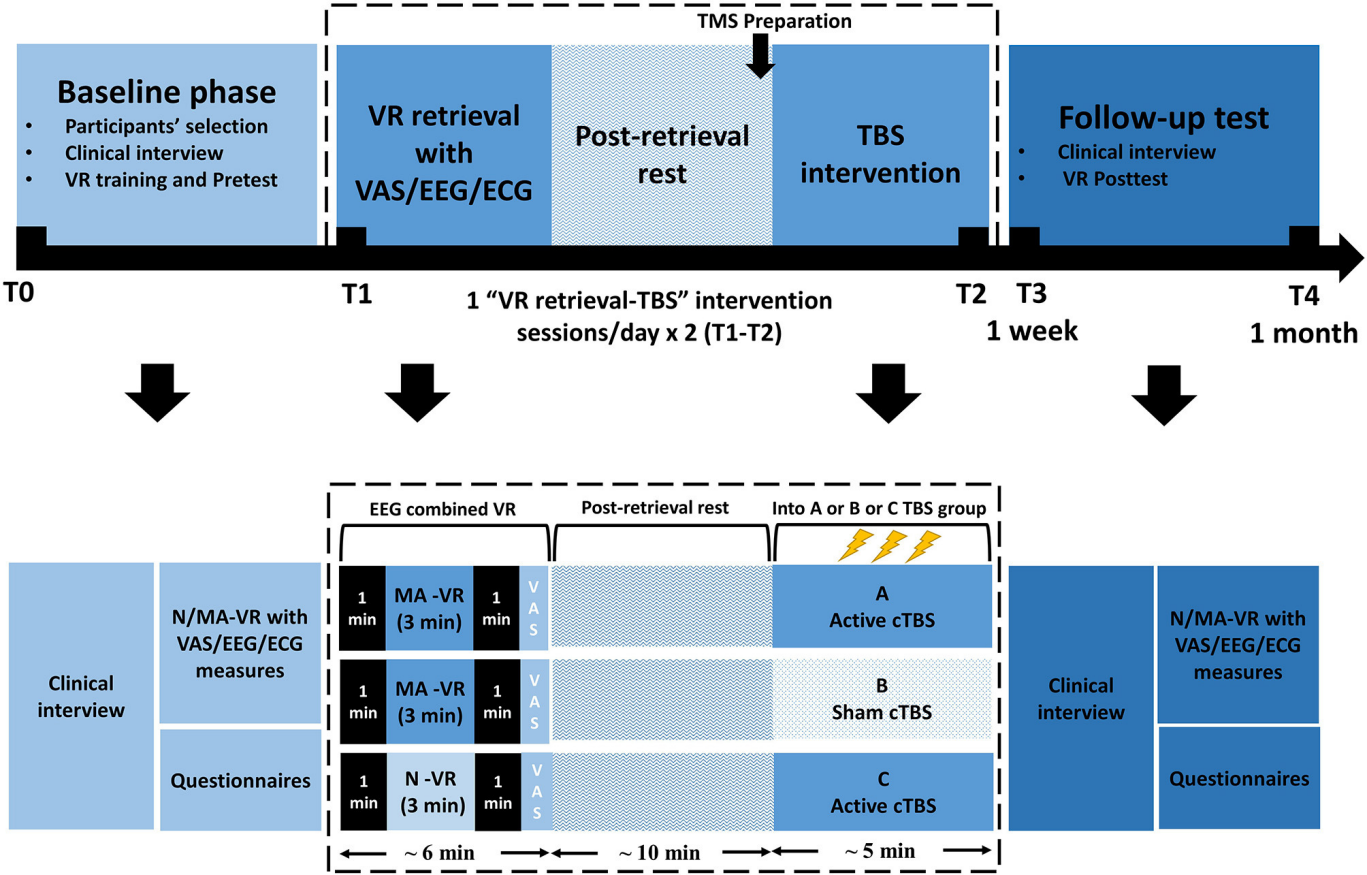
Participants' timeline. VR, Virtual Reality; cTBS, continuous theta-burst stimulation; N/MA-VR, neutral/methamphetamine scenes displayed by VR; VAS, visual analog scale; EEG, electroencephalography; ECG, electrocardiography.

This is a randomized, sham-controlled double-blind study. Participants will be randomly assigned by main investigator to three parallel groups using computer-generated random numbers with a 1:1:1 allocation ratio, either receive (1) MA-related cues retrieval in VR combined with active TMS (group A: MA VR scene + TBS) or (2) sham TMS (group B: MA VR scene + sham TBS), or (3) neutral cues retrieval in VR combined with active TMS (group C: neutral VR scene + TBS). Participants, outcome assessors, and data analysts will be blinded to the group assignment during treatment sessions. The doctors delivering TMS treatment will know the allocation sequence but will not be involved in the assessment. Once treatment is completed, participants will be asked to guess the specified intervention they have received to determine the actual effect of TBS. Then the clinical evaluator will debrief the participants about the study procedures. The two “VR retrieval–TBS” treatment sessions will be scheduled in 2 days within 1 week using an MA-related or neutral VR scene. Clinical, psychological questionnaires and EEG will be carried out at baseline (T0), each treatment session (T1/T2), the week of the last treatment (T3) and over 1 month after treatment (T4), as shown in Figures 1, 2. This protocol follows SPIRIT recommendations.

### Study Setting

This clinical trial will be conducted in Shanxi Drug Rehabilitation Center (Taiyuan, Shanxi Province, China) involving the “VR retrieval–TBS” sessions and a series of assessments (as described in outcome measures).

### Participants

Participants with MUD in Shanxi Drug Rehabilitation Center will be voluntarily recruited to this treatment. After initial confirmation, eligible participants will then be required to sign an informed consent. The number and reasons of exclusion individuals, as well as those who reject or withdraw consent will also be recorded. *Inclusion criteria:* Selected participants will have to (1) be clinically diagnosed with Amphetamine-Type Substance Use Disorder according to Diagnostic and Statistical Manual of Mental Disorders (DSM-5) criteria, (2) abstinence periods at least 2 weeks without obvious withdrawal symptoms (e.g., drowsiness and dysphoria), (3) be aged 18 and 45 years old, and be right-handed, (4) be able to speak and read Chinese, (5) sign a consent form before intervention procedure.

*Non-inclusion criteria:* Participants meeting at least one of the following criteria will not be enrolled in this study: (1) meeting the diagnostic criteria of any other psychiatric disorder under DSM-5; (2) using other kinds of drugs (e.g., heroin, cocaine) in the past 30 days; (3) having contraindications to TMS treatment (head trauma, epilepsy or the history of epilepsy, metal implant, pregnancy and so on); (4) having a history of mental illness or a family history of mental illness; (5) be insensitive to VR scene (identified at the baseline); (6) having received psycho-therapy or TMS intervention in the last 6 months; (7) receiving medication treatment recently; (8) illiteracy; (9) having visual or hearing problems; (10) the individual threshold of TMS was higher than 50% of the maximum output of the TMS equipment; (11) scheduled to receive psycho-therapy or medication treatment for the next 2 months. *Exit criteria:* Participants will be allowed to withdraw from the clinical trial if they (1) voluntarily wish to stop the TBS sessions; (2) can hardly tolerate any aversive reactions (e.g., itching, numbing) or have a desire for other treatments; (3) do not complete the TBS sessions for other reasons; (4) suffer from worsening symptoms such as enhanced craving.

### Intervention

The “VR retrieval–TBS” intervention will be given twice within 1 week. Each intervention session contains a MA-related VR scene to retrieve memory (or a neutral VR scene in a control group), a 5-min TBS treatment, and a 10-min interval in between them to conform with the suggestion that a 10-min reconsolidation window may improve the effectiveness of the subsequent intervention (8, 57). A short TBS trial will be delivered before the full-length treatment to decrease risks and improve clinical adherence. The pulse intensity of TBS will be assigned according to identified individual resting motor threshold (RMT).

#### VR Retrieval

Two types of VR scenes containing a series of neutral or drug-cues respectively will be presented in this study. As introduced in our previous study (43), the VR neutral scene for VR interaction practice at the first time that the participants using VR devices will be, a room with a desk, two spheres, two cubes, two cylinders, a laptop, and a visual analog scale (VAS) presented on the wall of the room. The spheres, cubes and cylinders are interactive, which means they can be picked up or stacked in the VR environment using the handle. This scene for practice will be presented once for 5 min. Another neutral scene will be presented in the baseline test and post-test. The neutral scene will be presented for 3 min followed by MA scenes after a 1-min interval of darkness in between. This neutral scene is a room with a sofa, a coffee table, and a standing lamp. There are about 50 triangle, square, or cylindrical blocks on the coffee table, about 10 cm in diameter. All the blocks are interactive. There are two MA scenes. Both scenes will be presented in the baseline test and the post-test, each 3 min, with a 1-min interval in between. The first MA scene is a room with a sofa, a coffee table, a standing lamp, a wardrobe, and a cabinet. There are many items on the coffee table, including a small bag of MA, a roll of foil, a piece of foil, lighters, plastic beverage bottles, paper money, straws, tools for diluting and filtering MA (a beverage bottle with straws and filters), a cellphone and a laptop. A video of playing a card game is played on the laptop screen. There is a TV hanging on the wall, which can play dance music or gambling game videos through the VR handle. The second MA scene is a room with a bed, a coffee table, and a chair. The items on the coffee table are similar to those in the first MA scene. There is a male or female avatar on the bed doing a series of actions of taking MA. All sofas or beds in the virtual scene have seats of similar heights at the corresponding positions in the real room. The MA-related VR scenes that we would like to use have been validated for their effect on MA memory retrieval based on the data in our lab and previous studies (58). In the baseline test and post-test, after the three scenes (neutral, MA1, MA2), the VAS is presented on the wall of a room in VR. Two groups (MA VR scene + TBS, MA VR scene + sham TBS) will view either the first or second MA scene prior to TBS treatments. Another group (neutral VR scene + TBS) will view a neutral VR scene before TBS treatments. Each scene for memory retrieval will last 3 min at each treatment session with 1 min pre-dark and 1 min post-dark, ended by an embedded VAS for self-reported craving rating. Real-time EEG for brain activity will be recorded while the presentation of VR scene (see *Outcome Measures*).

All VR scenes will be displayed by HTC VIVE VR system containing a wired headset (110 degrees, 1,080 × 1,200, 90 Hz) and a wireless handle controller to provide participants with direct, realistic interactions. With a head-mounted display, participants wearing a headset will immerse into the VR scenes. The wireless controller will also be accessible for participants to interact with objects in the VR scenes and rate VAS. The VR scenes will be monitored by a care provider on a desk computer (Alienware 15-R2748, i7-7700HQ 16G 256GSSD+1T GTX1070 8G discrete graphics FHD). These VR scenes have been developed by our collaborative team with unity 3D and used in our previous study (43).

#### TBS Protocol

##### Target to Stimulate

**Figure 3:**
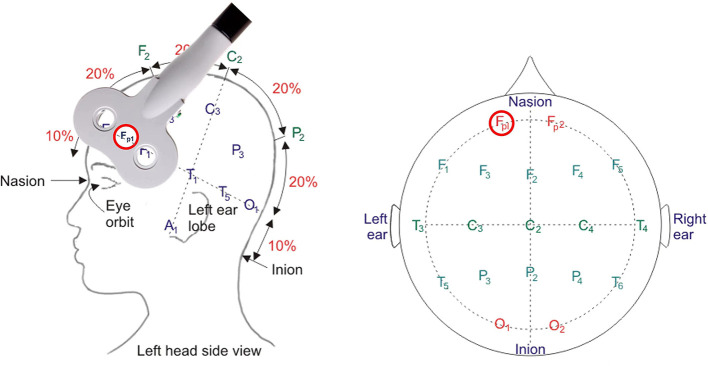
The stimulation coil targeting Fp1 on EEG International 10–20 system positioning.

Refer to related research and large-scale Magnetic Resonance Imaging/functional Magnetic Resonance Imaging (MRI/fMRI) data from https://neurosynth.org/, the OFC, functioning (including both orbital and media area) with encoding specific stimuli value (59, 60) has been located with an averaged MNI coordinates (-4, 36,−18). Following previous suggestion (61), the stimulated site targeting OFC will be located according to the standardized international EEG 10–20 position (Fp1). This brain area will be labeled on an EEG 10 - 20 system cap. The coil will be placed tangent to the scalp at the contact point (Figure 3). During the TBS or sham treatment, the TMS coil over Fp1 will be fixed by a holder on the coil's handle during the treatment. We will pause to adjust the position if there is any movement of the coil or head according to the participant's feedback.

##### TBS Pattern

The cTBS will be applied to reduce activation of the OFC circuit. TBS sessions will be carried out with a hand-held figure-of-eight coil with the size of 90 mm diameter of one half of the “8” shape (B9076, Yiruide Co., Wuhan, China), attached to a YRD CCY-I stimulator (Yiruide Co., Wuhan, China). Following a 10-min rest after memory retrieval, participants either in the MA VR scene + TBS group (*n* = 15) or in the MA VR scene + sham TBS group (*n* = 15) or in the neutral VR scene + TBS group (*n* = 15) will receive two TBS sessions within 1 week. In each session, all participants will receive two 1,800-pulse trains of cTBS in 5 min (three pulses are given at 50 Hz as one burst; a burst will be given every 200 s as 5 Hz; one train for 120s, containing 1,800 pulses) with a pulse intensity of 80% of RMT following Hanlon et al. (62)'s suggestion. A 60-s break will give to participants after the first train (1,800 pulses). The choice of TBS intensity will consider the participants' real-time feedback and health condition to TMS stimuli. If a participant reported unbearable discomfort with TBS stimulation, we will turn down the intensity gradually until acceptable. If a participant still reported unbearable discomfort when the stimulation intensity was lower than 60% of the RMT, this participant will withdraw from the experiment. These parameters have been applied by previous studies (62) and proposed in safety guidelines (63).

##### Intensity Identification

The RMT is suggested as the minimal TMS intensity required to elicit motor evoked potentials (MEPs). It will be identified by flexion/extension movements of any finger, especially the index and little finger, in at least 5 of 10 consecutive MEPs trials (32). A single TMS session will be conducted by a figure-of-eight TMS coil following previous suggestions (64), with a gradual increase (5% each time) from 40% of median output intensity up to an intensity which could induce slight but significant finger movement. The coil will be applied over a suggested “finger motor cortex,” with a TMS-matched elastic cap. This location was examined about approximately 8–11 cm lateral and 0–4 cm anterior to the cranial vertex (65). Thus, the TMS provider should concern with both cap-provided “marker” and information about a relative position, to find the “hotspots” accurately.

##### Sham TBS Protocol

The sham TBS treatment will be conducted with identical conditions of active TBS using a sham TBS coil. The sham coil has no difference in shape and appearance to that of the active coil except for an additional black coil sheath for reduction of the diffusion of magnetic fields. It delivers stimulation over only scalp skin and superficial muscles without proposed physiological modulation effects on the cortex (66).

### Outcome Measures

#### Primary Outcome Measure

We will evaluate the short-term effects of Retrieval-TBS treatment in the reduction of cue-induced drug craving and cue reactivity by comparing EEG in VR scene between baseline (T0) and post-test (T3).

***EEG*** will be recorded by a portable EEG system (Muse by Interaxon Inc.) during each VR session (3 min/scene × 1 MA-related VR scene and 1 neutral-related VR scene = 6 min) at T0 and T3, respectively. Each patient will wear an EEG headset before entering the VR scenes. Four channels (refer to EEG 10-20 system: FP1, FP2, TP9, TP10) and an original reference (FPz) will be retained to measure the brainwave spectrum including delta, theta, alpha, beta and gamma (with a sample rate of 256 Hz and 12 bit resolution), frequency bands between 45 and 60 Hz were omitted because muse hardware applied notch filters at 50 and 60 Hz to reduce environmental noise (67). According to a previous study, there was a significant difference between drug-cue and neutral-cue-induced EEG activities (68). In terms of drug-cue related memory, the frontal theta activity used as a feedback signal in adaptive regulation was highlighted for memory updating (69), which has been suggested as an indicator of the enhanced binding of incoming interconnected information in memory formation (70), while the beta activity which reflects a memory promoting state (70) indicated the level of memory integration in reconsolidation (71). Since the wearable devices available in VR have been applied to classify emotional states (72), we plan to use wearable EEG combined with VR scenes to observe the brain activity associated with memory in the current study. The average power in EEG frequency bands (i.e., δ = 1–4 Hz, θ = 4–8 Hz, α = 8–13 Hz, β = 13–30 Hz, γ = 30–50 Hz) are real-time computed in hardware and sent alongside an indicator of data quality from each sensor. The Muse manufacturer provided on-device computed FFTs (https://sites.google.com/a/interaxon.ca/muse-developer-site/museio) were used to estimate the spectral power density (μV2/Hz) for frontal electrodes in each frequency band in this study. EEG spontaneous measures at the frontal sites were calculated, as shown in Figure 2, by 1 min post-dark data minus 1 min pre-dark data in each frequency band in each VR scene. We used state-related EEG to calculate the EEG activities for drug-related craving state in VR, because cue-induced craving is related to a tonic, slowly changing craving state and Muse is a portable device that may not stably acquire EEG activities during walking in VR scene. Previous studies have used “state-related EEG” to compare different EEG activities in different state (56, 72, 73). Because of the non-normal distribution, power density value and ratios were log-normalized. For statistical tests, a two-tailed alpha of 0.05 was used unless explicitly stated otherwise.

#### Secondary Outcome Measures

The secondary outcome will focus on the long-term effects of repetitive treatments on drug-cue-induced response. Evaluations introduced in primary outcome will also be collected in the follow-up test (T4). Furthermore, self-reported craving in VAS as a subjective indicator for cue-induced drug craving, physiological parameter (cue-induced heart rate) and possible co-variables corresponding to substance-taking history, emotional and mental state will be measured with the clinical diagnosis and psychological questionnaires at baseline (T0), post-test (T3) and follow-up test (T4) respectively.

***VAS*** embedded in VR scenes is a 100-point visual analog scale ranging from 0 (no craving) to 100 (high craving) for self-reported craving. Each patient will be directed to evaluate their current craving at a black screen on one wall of a virtual room. They will be guided to focus on their momentary feeling to make an assessment. The VAS points at baseline (T0) will be compared with the post-test after the last treatment (T3) to identify an expected reduction in self-evaluated craving. The median, range, average and standard deviation of VAS will be calculated for analysis.

***Electrocardiography (ECG)*** Given the association of MA with autonomic nervous system dysfunction (74) and the significant difference in heart rate between drug-cue and neutral-cue in the cue-induced craving paradigm (75), ECG (BIOPAC ECG module, with sampling rate of 1,000 Hz) will be recorded in this experiment as an indicator of physiological changes. In addition, changes in the heart rate interval can reflect abnormalities of autonomic nervous function (76). In addition, its activities are related to executive functions such as working memory, attention maintenance, and response inhibition involved in prefrontal cortex activities (77). In MA studies, it shows significantly reduced heart rate variability in MA populations (76), and the difference between drug-cue and neutral-cue is significantly associated with subjective cravings (78). Therefore, ECG will be recorded at the same time with EEG.

***Anxiety*** as a specific unpleasant emotional state has been observed as a common psychiatric comorbidity in addicts (79). Each patient will be required to complete the Self-Rating Anxiety Scale (SAS) at T0, T3 and T4 (80). The questionnaire consists of 20 items rated on a 1-4 points scale, with a total score ranging from 20 (mild anxiety) to 80 (severe anxiety). The SAS has adequate internal consistency alpha coefficients (α > 0.7). It is a widely used measurement of anxiety levels both in research and in clinical practices.

***Depression*** is another common emotional state exhibited following intense drug craving (79). It will be assessed at T0, T3 and T4 with Self-Rating Depression Scale (SDS) (81). The questionnaire consists of 20 items rated on a 1-4 points scale, with a total score ranging from 20 (mild depression) to 80 (severe depression). The SDS has satisfactory alpha coefficients (α > 0.7) and it has been implemented widely in clinic trials.

***Mood State*** will be evaluated at T0, T3 and T4 with the Profile of Mood States -short form (POMS-SF) (82). The self-reported scale includes 40-items to identify the extent of feeling in respective mood state. The POMS-SF is indicated for current emotional state on a 5-point scale (1 = “very slightly or not at all” to 5 = “extremely”). It is widely used for clinical practice in China (83) and its accessibility and consistent reliability coefficients have been identified among Chinese (with *Cronbach's* α > *0.7*).

### Participant Timeline

All participants will be introduced to follow the schedule of baseline phase, treatment sessions and follow-up test. All patients passing the screening process will enter the treatment procedure. Participants will be grouped into A, B or C to receive MA-VR retrieval combined active TBS (MA VR-active TBS) or MA-VR retrieval combined sham TBS (MA VR-sham TBS) or neutral-VR retrieval combined active TBS (neutral VR-active TBS), respectively. The treatment includes two TMS intervention sessions within 1 week. Each session takes about 30 to 40 min including preparation time, VR retrieval of 3 min, relaxation of 10 min and continuous TBS of 5 min. The post-test will be conducted 1 to 4 days after the last session and the follow-up test will be taken over at least 1 month later. The timeline for participants in this trial is illustrated in Figure 2.

### Sample Size

According to Hanlon et al. (28) study, 25 cocaine users either receiving active- or sham- pre/post TBS treatment showed a significant difference in the reduction of cue craving (*F*_1, 96_ = 5.65, *P* = 0.02). The effect size (*f*= 0.2496) calculated by formula $r^{2}=\frac{df1^{*}F}{df1^{*}F\;+\;df2},f=\sqrt{\frac{r^{2}}{{1-r}^{2}}}$, the 3 (between factor: MA VR-active TBS vs. MA VR-sham TBS vs. neutral VR-active TBS) × 4 (within factor: four repetitive tests) design of the current protocol, the proposed alpha error (α = 0.05) and power (β = 0.95) will be used as the parameters for a prior sample size calculation (in G.Power 3.1). The required sample size is 45 in terms of repeated measures ANOVA with within × between interaction effect.

### Data Collection and Statistical Methods

The screener will ensure the inclusion and exclusion criteria and collect basic characteristic data before randomization. The outcome evaluators will play an important role in measurements of the primary and secondary outcomes. The data of all participants will be stored in documents and kept in locked boxes by the main investigator to protect personal information. Furthermore, the EEG data will be stored by the primary research on a locked computer with a password.

#### Data Preparation and Descriptive Statistics

After inspecting original data, identifying unusual cases, transforming and summarizing data-sets according to different types of variables: the mean, median, standard deviation, ranges and valid samples will be required at first, as well as the counts and percentages specific for categorical variables. Firstly, running SPSS 21.0 for basic check of validated data both based on the extent of treatment completion (>85%) and accepted level of missing/obscure/contaminated data (<10–20%). The missing data will be estimated via multiple imputations. Secondly, the distribution of data will be analyzed both by stem-and-leaf plot (SPSS 21.0), the actual data calculated for normality overlay a normal curve will be required to determine that to what extent it is a normal distribution (without significance at given alpha = 0.05). For a poor fitted normal curve, non-parametric inferential will be selected, while the standard or similar standard alloy will run the parametric inferential.

#### Inferential Statistics to Determine the Efficiency of Intervention

The statistical significance (sig) and effect size (ES) will be evaluated for treatment-effects determination either by paired *t*-test and repeated measures ANOVA test [two factors, test time (baseline vs. post-test) and treatment (MA VR scene + TBS vs. MA VR scene + sham TBS vs. neutral VR scene + TBS)] in parametric estimations or Friedman's chi-square and Kendall's W coefficients in non-parametric statistics. These differences between baseline and post-tests will be compared firstly between the three parallel groups for self-reported craving and neurophysiological measures (e.g., EEG and ECG).

#### Regression Analysis for Variables Prediction

A generalized linear modal (GLM) will be used for regression analysis of co-variation (e.g., emotion and mood state) and of the follow-up long-term effects by SPSS 21.0. The selected model with different statistics will be specified based on data characteristics such as the type of distribution. For example, a multiple linear regression model will be introduced according to stepwise regression to test whether there was any potential influencing factor among secondary indicators.

### Monitoring

#### Definition of Adverse Events and Potential Risks

(1) referring to previous neuro-modulation studies, rare cases reported adverse effects of TMS, although some tolerable but uncomfortable feelings were reported by participants, such as mild itching, numbness or tingling, a slight headache, or head discomfort. However, these symptoms only existed for a short time and were relieved after stimulation ceased;

(2) according to existing cases and clinical practices, patients with following conditions are at higher risk to experience adverse sides in the treatment: (1) experiencing epileptic seizures or whose immediate family member has a history of epilepsy and mental illness; (2) having a history of brain diseases such as brain trauma, cerebral hemorrhage, cerebral infarction, or intracranial infection; (3) having intracranial metal or other foreign bodies;(4) having a pacemaker placed in the body.

#### Identification Method and Management System

(1) above potential risks will be considered within exclusion criteria to select adequate participants at the screening stage. These criteria will be re-confirmed again before the delivery of stimulation;

(2) individualized parameters for treatment: the intensity will be identified as RMT according to the individual threshold, and it will be adjusted (80–110% RMT) based on the tolerance of each patient;

(3) if there is persistent pain, intolerable, or the participant subjectively requests to withdraw, the treatment shall not be continued and concomitant care will be offered.

## Discussion

This protocol is proposed for a clinical translational study based on the theoretical framework of memory reconsolidation, using immersive VR for memory retrieval and TBS for neural modulation. The main purpose of this study is to investigate the effectiveness of neuromodulation during the time window of drug-related memory reconsolidation to reduce the craving of MA-dependent patients.

It has been proved in many clinical studies that the effect of pharmacological or behavioral intervention within the reconsolidation time window of drug-related memory is much better than that of intervention outside the reconsolidation window (84, 85). The enhancement of memory intervention during reconsolidation is due to the elevated lability during the post-retrieval period when the original memory trace is putatively re-written by pharmacological or behavioral interference (42). Non-invasive brain stimulation methods such as TMS and transcranial direct-current stimulation (tDCS), which have been applied to clinical treatment of drug addiction in recent years (62, 79), provide another therapeutic approach to intervening in the reconsolidation process. In addition, the TBS, a newly developed repetitive TMS pattern, is characterized by its short duration of application but long-term and better therapeutic effect (40, 86). We select the OFC as the target of TBS intervention because of the reciprocal connection between the OFC and the BLA which regulates the encoding and updating of the incentive salience of reward cues in associative memory (27, 87, 88). Evidence from pre-clinical studies shows that disrupting the activity of the OFC-BLA circuit during reward learning or memory retrieval results in impaired cue-induced reward seeking response (27) by interrupting the encoding or updating process of incentive salience (59, 89–92). Therefore, we postulate that TBS on the OFC may disrupt or weaken the functional connection between the OFC and the BLA during MA-related memory, thereby it can reduce the motivational and emotional value of drug cues and the craving response when an MA-dependent patient encounters cues.

Furthermore, the effect of reconsolidation-based interventions relies on the level of memory reactivating (93, 94). The more vivid the retrieved memory is, the better effect of intervention during the reconsolidation should be. A prominent advantage of VR in memory reactivation is the sense of presence generated by interactive, diverse cues presented in immersive and vivid scenarios (95, 96). MA-dependent patients are more likely to re-experience their drug-related episodic memories and generate MA cravings encountering drug-related cues and context in VR (95). Therefore, we propose that the incorporation of VR scenes in memory reactivation will further improve the effect of the follow-up TBS intervention.

Some caveats should be considered in the interpretation of findings from this study. First, the study will be conducted in compulsory drug rehabilitation centers where magnetic resonance imaging cannot be performed. Therefore, we will be unable to navigate precisely to deliver stimulation to a specific brain site nor to accurately evaluate the effect of the TBS intervention on the functional connectivity of the OFC-BLA circuit with the aid of fMRI. An alternative resolution is to assess the effect of TBS with EEG resting-state analysis before and after treatment (91, 97). In addition, our alternative plan is to locate the stimulation site by an EEG 10-20 cap (98). Although the location for delivery will be marked on the cap, it is difficult to remain stable across multiple treatment sessions. A portable neuro-navigation system for TMS is highly expected to improve future clinical practice.

The current study combines post-retrieval TMS intervention with VR to develop a new reconsolidation-based treatment for MUD patients. The findings of this study may provide the first and compelling evidence that TMS modulation after VR retrieval can reduce self-reported craving and drug-related cue reactivity. It will not only provide new ideas and insights for the clinical intervention of MUD, but also improve the understanding of the neural circuit mechanism of the reconsolidation-based intervention. Particularly, the TBS protocol, a highly effective stimulation pattern, may improve the efficiency of clinical intervention of TMS and promote the clinical application of therapeutic neuromodulation.

## Ethics Statement

The Ethics Committee of the Institute of Psychology, Chinese Academy of Sciences has approved this protocol (H19007). The study will be carried out in accordance with the recommendations of this committee. All participants will sign an informed consent form (which will be gathered by the main investigator), providing their wish to do so, in accordance with the Declaration of Helsinki and with national and local regulations. The study is registered in the Chinese Clinical Trial Registry (www.chictr.org.cn) with ID: ChiCTR1900026902.

## Author Contributions

YW and YL conceived the study and designed the study protocol. YW wrote the primary manuscript and is responsible for formal analysis. XD, XC, and SQ contributed to the further development of the manuscript. YL and LW applied for funding. YL, XD, LW, and FJ provided supervision and resources. YW, YL, and LW prepared the ethical review application. XH is the leader of the investigation. XH, XD, QL, FeW, XY, and FaW are responsible for enrollment, data collection, intervention, and validation. SQ is responsible for data curation. All authors read and approved the final manuscript.

## Funding

This work was supported by Chinese National Programs for Brain Science and Brain-like Intelligence Technology (2021ZD0202104), CAS Engineering Laboratory for Psychological Service (KFJ-PTXM-29), National Natural Science Foundation of China (32020103009), and Beijing Municipal Science and Technology Commission (Z171100000117014).

## Conflict of Interest

The authors declare that the research was conducted in the absence of any commercial or financial relationships that could be construed as a potential conflict of interest.

## Publisher's Note

All claims expressed in this article are solely those of the authors and do not necessarily represent those of their affiliated organizations, or those of the publisher, the editors and the reviewers. Any product that may be evaluated in this article, or claim that may be made by its manufacturer, is not guaranteed or endorsed by the publisher.

## Abbreviations

BLA: basolateral amygdala
EEG: electroencephalography
MRI/fMRI: Magnetic Resonance Imaging/functional Magnetic Resonance Imaging
MUD: Methamphetamine Use Disorder
OFC: Orbitofrontal Cortex
TMS: Transcranial Magnetic Stimulation
TBS: Theta-Burst Stimulation
VR: Virtual-Reality
VAS: Visual Analog Scale.
